# Investigating the influence of gamification on motivation and learning outcomes in online language learning

**DOI:** 10.3389/fpsyg.2024.1295709

**Published:** 2024-05-27

**Authors:** Zijun Shen, Minjie Lai, Fei Wang

**Affiliations:** ^1^Department of Foreign Languages, Sichuan University of Media and Communications, Chengdu, Sichuan, China; ^2^International College, Guangzhou College of Commerce, Guangzhou, Guangdong, China; ^3^The University of Sydney, Darlington, NSW, Australia

**Keywords:** gamification integration, learners' motivation, learning style preference, language learning outcomes, online language learning

## Abstract

**Introduction:**

This study investigates the influence of gamification integration on language learning achievement among Chinese students while probing the mediating role of learners' motivation. Furthermore, it extends the boundaries of this investigation by exploring the moderating effect of digital literacy as a psychological predisposition.

**Methods:**

Data is collected through surveys from Chinese students enrolled in linguistic programs, employing a stratified random sampling technique and analyzed via SmartPLS SEM.

**Results:**

The findings affirm the significant and positive impact of gamification integration on language learning achievement. The study introduces a moderated mediation model where learners' motivation serves as the mediator, and digital literacy acts as a moderator, further accentuating the significant impact of this integrated approach.

**Discussion:**

This research advances our theoretical understanding of language learning, validating gamification's effectiveness as a motivational tool, and introduces digital literacy as a critical factor, providing deeper insights into personalized language learning experiences.

## Introduction

Gamification, defined as “the incorporation of game-like elements and principles into non-game contexts” (Saleem et al., 2022), has emerged as a vital concept in modern education and beyond (Sailer and Homner, 2020; Krath et al., 2021). Gamification represents a multifaceted approach that intertwines motivation, engagement, and learning, and its significance is underscored by its burgeoning relevance in contemporary pedagogical and technological landscapes (Krath et al., 2021). Numerous scholars including Manzano-León et al. (2021) and Hamari (2019) have outlined key game elements, including points, badges, leader boards, and narratives, which are harnessed to drive participation and enthusiasm. Recent theorization and application of gamification reveal a dynamic field that continues to evolve and integrate in a wide array of educational spheres. In this perspective, Luarn et al. (2023) study aligns with self-determination theory and emphasizes the role of autonomy, competence, and relatedness in fostering intrinsic motivation through gamified experiences. Moreover, the application of gamification is not only showcased in traditional educational settings but also in corporate training (Wang et al., 2022) and healthcare (Damaševičius et al., 2023), reflecting its widespread adaptability across domains.

The relationship between gamification integration and language learning outcomes is of paramount importance, particularly in the context of language education's perennial challenge of sustaining learners' motivation and engagement. Recent studies (e.g., Luarn et al., 2023) accentuate the positive influence of gamification on motivation, with its potential to enhance the quality of learning experiences. In language learning, where perseverance and dedication are essential, gamification offers a promising means to mitigate attrition rates and reinforce proficiency development. Studying the relationship between gamification integration and language learning outcomes is crucial as it has the potential to revive learners' enthusiasm, enhance their language skills, and equip them for global communication demands in our interconnected society.

In addition, the study elucidates the underlying mechanisms through which gamification integration influences language learning outcomes: learners' motivation. While it is evident that gamification can stimulate motivation and engagement in learners, the precise channels through which this heightened motivation translates into improved language proficiency require deeper exploration (Luarn et al., 2023). We rely on the self-determination theory (Deci and Ryan, 1985) to predict the link between gamification integration and language learning outcomes, mediated by learners' motivation. By introducing learners' motivation as a mediating variable, we aim to uncover the intermediary processes that mediate the effect of gamification on language learning outcomes. Understanding this mediating mechanism is imperative for gaining a more comprehensive understanding of the underlying associations and for providing educators and instructional designers with valuable insights to optimize the integration of gamification elements in online language learning platforms.

In spite of the significant progress achieved in comprehending the mediating role of learners' motivation in the gamification-language learning nexus (Krath et al., 2021; Saleem et al., 2022), a notable research gap persists concerning the moderating influence of individual differences, particularly learning style preferences. We anticipate that learning style preferences, which reflects a psychological predisposition construct (Karthigeyan and Nirmala, 2013), may significantly influence how learners respond to gamification elements. This assumption is anchored on the attention restoration theory (Kaplan et al., 1989), which posits that individuals exhibit varying cognitive responses to environmental stimuli. Applying this theoretical lens, we postulate that learners with different learning style preferences may interact differently with gamified language learning environments, subsequently affecting their motivation and, ultimately, their language learning outcomes. Thus, by incorporating learning style preference as a moderating variable, our study addresses this critical research gap and provides a more nuanced understanding of the interplay between gamification, motivation, and language learning outcomes in a diverse learner population.

## Literature review

### Self-determination theory

The Self-Determination Theory (SDT) is a widely recognized framework within the fields of psychology and education that provides a comprehensive perspective on human motivation and behavior. According to Deci and Ryan (1985), the SDT proposes that humans possess inherent psychological requirements for autonomy, competence, and relatedness. These requirements are essential factors that significantly influence human motivation and overall wellbeing.

The fundamental concept of SDT is the differentiation of many forms of motivation. These include intrinsic motivation, which refers to the engagement in an activity for its inherent satisfaction, and extrinsic motivation, which is driven by external variables such as incentives or punishment. The SDT places significant emphasis on the cultivation of intrinsic motivation due to its positive correlation with increased levels of perseverance, involvement, and optimal educational achievements (Deci and Ryan, 2012). The SDT has been extensively utilized across many domains such as education, healthcare, and employment environments, with the aim of fostering motivation, wellbeing, and individual development. Hence, this theoretical framework serves as a fundamental basis for comprehending the principles behind designing environments and interventions that foster intrinsic motivation, resulting in behaviors and outcomes that are more sustainable and rewarding. In our study's context, the theory specifically elucidates how the integration of gamification promotes a sense of autonomy, competence, and relatedness, hence stimulating learners' motivation and resulting in positive language learning results.

### Attention restoration theory

Attention Restoration Theory (ART), introduced by Kaplan et al. (1989), provides a theoretical lens to understand the potential moderating role of individual differences, such as learning style preferences, in the context of gamified language learning. ART purports that environments that allow individuals to effortlessly direct their attention can facilitate cognitive restoration and stress reduction. These environments are often characterized by features like natural settings, fascination, and a sense of coherence.

In the context of gamified language learning, ART can be applied to suggest that learners with different learning style preferences may have varying cognitive responses to the gamified elements. For instance, individuals with a visual learning style might find gamification elements that include visual stimuli, such as images or graphics, more captivating and conducive to attention restoration. Conversely, those with an auditory learning style may respond differently to auditory cues embedded in the gamified experience. By integrating ART into the research framework, we can explore how these individual differences in cognitive responses to the gamified environment may moderate the relationship between gamification, motivation, and language learning outcomes, providing valuable insights for personalized learning experiences.

## Theoretical framework

### Relationship between gamification integration and language learning outcomes

Research in the field of language learning has increasingly explored the relationship between gamification and language learning outcomes (Krath et al., 2021), shedding light on the potential benefits of integrating game elements and principles into language education (Sailer and Homner, 2020). According to Saleem et al. (2022), gamification has emerged as an effective motivator in language learning. One fundamental aspect of gamification is its incorporation of game-like elements and mechanics into non-game contexts, including educational settings (Mee Mee et al., 2020). Research conducted by Panmei and Waluyo (2022) endorsed that the gamification strategies in the language learning context have the potential to motivate learners and significantly enhance their engagement in language learning tasks. Moreover, in a recent meta-analytical study (Huang et al., 2020) verified that by integrating features like multiplayer games and discussion forums, learners can engage in meaningful language exchanges, mimicking real-world language use and thus improving their language proficiency. Furthermore, the effectiveness of gamification in language learning has been linked to the development of specific language skills. For instance, Panmei and Waluyo (2022) argued that game-based approaches can be particularly beneficial for enhancing vocabulary acquisition and retention, as games often require learners to use new vocabulary in context repeatedly. Similarly, the research conducted by Kayimbaşioglu et al. (2016) demonstrated that mobile language learning apps employing gamification strategies can provide learners with flexibility, allowing them to engage in language learning at their own pace and in various settings, thus nurturing language learning outcomes.

### Mediating role of learners' motivation

In addition to examining the direct relationship between gamification integration and language learning outcomes, the study projects the mediating role of learners' motivation in reinforcing the underlying association. Drawing on the SDT (Deci and Ryan, 1985), scholars have emphasized that intrinsic motivation, which arises from genuine interest in the learning process, is a crucial factor for sustained engagement and enhanced learning outcomes. Gamification, through the incorporation of game elements such as points, badges, and leader boards, has been shown to foster this intrinsic motivation (Xu et al., 2021). These elements create a sense of achievement and competition, fueling learners' enthusiasm and persistence in language learning tasks (Chapman and Rich, 2017). Further, the influence of gamification on language learning outcomes is multifaceted. For example, gamified language learning platforms often feature interactive exercises and challenges that encourage active learning and the application of language skills in context. Prior scholarly works, such as Yasin and Abbas (2021) and Falah et al. (2021) indicate that such platforms lead to improved language proficiency compared to traditional methods. This is because, the immersive nature of gamification, facilitated by storytelling and narrative-driven scenarios, enhances comprehension and retention of language content (Hossein-Mohand et al., 2021). Subsequently, gamification can serve as an effective pedagogical tool for enhancing language learning outcomes by promoting active engagement and meaningful learning experiences (Kabilan et al., 2023). In the related stream, Roosta et al. (2016) corroborated that personalization has emerged as a significant aspect of gamified language learning. Personalized gamified environments, adapting content and challenges based on learners' performance and preferences (Urh et al., 2015), align with the principles of the SDT (Deci and Ryan, 2012). These gamified environments provide learners with autonomy, enhancing their motivation and engagement (Roosta et al., 2016). Besides, personalization accommodates the diverse learning styles and preferences of language learners (Urh et al., 2015; Xiao and Hew, 2023). This approach optimizes engagement and, subsequently, language learning outcomes by tailoring the gamified experience to individual learners.

### Moderating role of language style preferences

Language learning preferences refer to “individual inclinations toward specific learning styles, such as visual, auditory, or kinesthetic, in the process of acquiring a new language” (Riazi and Riasati, 2008). These preferences influence how learners engage with and absorb language content. Visual learners tend to favor graphical aids, charts, and written materials, while auditory learners prefer audio cues, spoken language, and dialogues (Schmidt and Watanabe, 2001). Kinesthetic learners, on the other hand, thrive in hands-on, interactive experiences that involve physical engagement (Xodabande, 2018).

The moderating role of language style preference in the context of gamification integration and its influence on language learning outcomes is a novel avenue for investigation. Recent research suggests that learners' cognitive responses to gamified elements may be influenced by their learning style preferences (Dantas and Cunha, 2020). For instance, individuals with a visual learning style may find gamification elements that include visual aids and graphics more captivating and conducive to attention restoration. Conversely, learners with an auditory learning style might respond more favorably to gamified activities that incorporate audio elements (Malik, 2019). Besides, kinesthetic learners, who thrive in interactive environments, may engage differently with gamified language learning platforms (Rozi et al., 2020). There exists empirical evidence suggesting that students pursuing science fields tend to exhibit a greater inclination toward collaborative learning, whilst students in humanities tend to exhibit a preference for auditory and individual learning approaches. Similarly, it has been observed that older students tend to exhibit a preference for the kinesthetic learning style due to their inclination toward hands-on learning experiences (Rozi et al., 2020). Another study conducted by Payaprom and Payaprom (2020) on marketing students demonstrated that these students have a preference for visual learning over auditory learning. These arguments render support for the moderator effect of learning style preference in underpinning the linkage between gamification integration and language learning outcomes via learners' motivation.

### Purpose and research hypotheses

**Figure 1:**
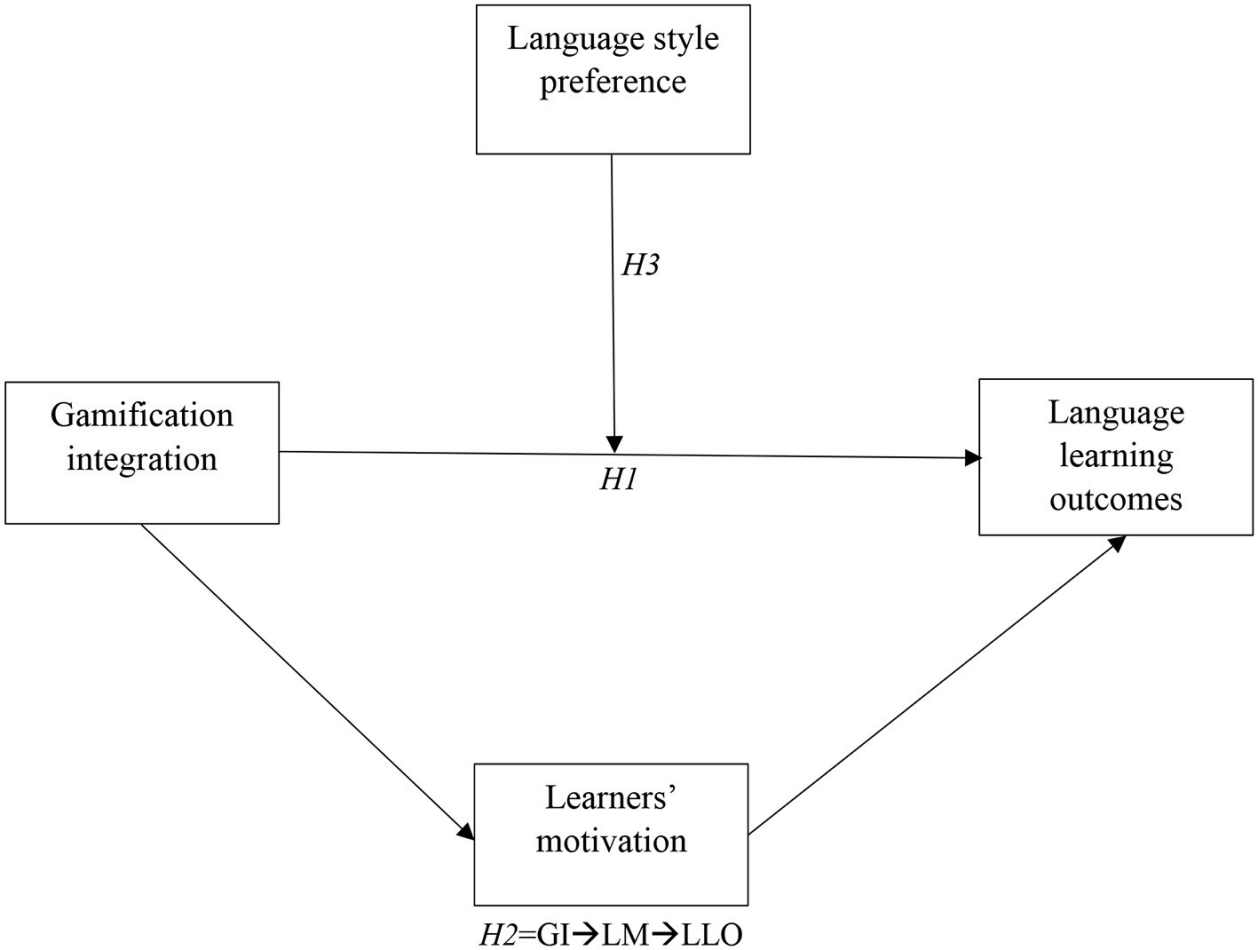
Conceptual model.

The purpose of this research is to explore the interplay between gamification integration and language learning outcomes, particularly focusing on the impact of learners' motivation and learning style preferences in this context. This study seeks to illuminate how gamified elements in language learning platforms influence student achievement and engagement, considering the roles of motivation and individual learning styles (Figure 1). In pursuit of this aim, we are proposing the following hypotheses:

Hypothesis 1. There is a significant relationship between gamification integration and language learning outcomes.

Hypothesis 2. Learners' motivation mediates the association between gamification integration and language learning outcomes.

Hypothesis 3. Learning style preference moderates the relationship between gamification integration and language learning outcomes, such that the association is more potent at higher levels of learning style preference.

## Method

### Sample and procedure

The current study examines the impact of gamification integration on language learning outcomes among Chinese students through the mediating role of learners' motivation and the moderating role of learning style preference. The research focused on Chinese students who were currently enrolled in linguistic programs. In order to achieve a sample that accurately represents the population, a stratified random sampling methodology was employed. The strata were established by considering factors such as age, gender, and linguistic proficiency levels, which were chosen to encompass the varied characteristics of the student population. To get a balanced representation of the characteristics, a random selection of students was conducted within each stratum. This ensured that the final sample encompassed a diverse range of individuals.

The process of data collecting was conducted by utilizing a survey strategy. Five-hundred participants were invited to participate in online surveys aimed at collecting data with respect to gamified online language learning platforms, motivation levels, learning style preference, and perceived outcomes in language learning. The survey was administered using established scales to assess gamification integration, language learning outcomes, motivation levels and preferences for learning styles.

To ensure data reliability and minimize potential biases, the survey instruments were rigorously designed and pilot-tested with a small group of participants before the main data collection phase. The survey was administered electronically to facilitate efficient data collection and minimize response errors. Moreover, multicollinearity assessment test was conducted to obtain variance inflation factor (VIF) scores to ensure that the study doesn't suffer from the common method biasness (CMB). The analysis yielded values < 3.3, ensuring no potential CMB threats.

### Participants

A total of 413 responses were analyzed using SmartPLS SEM. The study's demographic profiles encompassed a diverse group of Chinese students enrolled in linguistic programs. Twenty-nine percent of the participants fell into the 18–24 age group, around 46% of the participants belonged to the 25–34 age group, and 20% were in the 35–44 age group. The remaining 5% consisted of participants aged 45 and above. In terms of gender distribution, 53% of the participants were female, and 47% were male. To account for linguistic proficiency, the study included participants at various levels. Thirty percent of the participants were at the beginner level, 38% were at the intermediate level, and 32% were at the advanced level.

### Measures

The study utilized established scales adapted from prior literature to gather data for the survey. The questionnaire items were assessed using a 5-point Likert scale, with “1” indicating complete disagreement and “5” indicating complete agreement. Research instrument is illustrated in Appendix I.

### Gamification integration

The current study utilizes the research instrument utilized in previous studies, such as Xi and Hamari (2020) and Luarn et al. (2023) to assess gamification integration. The instrument comprises a total of 13 items, e.g., they were asked to rate how their level of “how critically you think when learning with these features.”

### Learners' motivation

We employed a research instrument originally devised by Ryan and Connell (1989) to assess learners' motivation. This instrument comprises a set of 5 items. Example item included “because I find them interesting and enjoyable.”

### Language learning outcomes

To measure language learning outcomes, the study adapted research instrument utilized by North and Schneider (1998) and Kahakalau (2017), which consisted of 6 items. For example, “I am proficient in speaking the target language” and “I consider myself moderately proficient in understanding spoken language in the target language.”

### Learning style preference

For measuring learning style preference, the authors borrowed questionnaire from Karthigeyan and Nirmala (2013), consisting of 15 items. The sample items included “I prefer listening lecture than reading textbook” and “I prefer learning by doing exercises and drills in the class.”

## Results

### Data analysis

The data in this study was analyzed using the “partial least squares structural equation modeling” (PLS-SEM) technique, and the PLS method was executed using SmartPLS (Version 4.0). There are several justifications for selecting PLS-SEM, often known as variance-based SEM. First, this study aims to investigate the extent to which exogenous variables account for the variance observed in the endogenous variables (Hair et al., 2018). Second, the study proposes a complex model and utilizes the SEM technique to assess the moderated mediation model (Henseler and Fassot, 2010).

### Measurement model

The researchers postulated a reflective framework in the present investigation. To assess the reflective measurement model, this study examines its internal consistency as well as its convergent and discriminant validity. The study evaluates the metrics of composite reliability (CR) and Cronbach's alpha to determine the internal consistency (Hair et al., 2018). Appendix I illustrates that all the values for CR and Cronbach's alpha above the minimum threshold value of 0.70 deemed acceptable. Moreover, for the purpose of assessing the convergent validity, the research employs the average variance extracted (AVE) and outer loadings, considering the minimum threshold of 0.50 (Hair et al., 2018). Further, the Appendix I displays the AVE and outer loading values, all of which surpass the threshold of 0.50. This observation confirms the presence of convergent validity within the study.

Furthermore, the present study also assesses the discriminant validity in order to ascertain that the correlations across different constructs do not surpass the correlations within the same construct (Hair et al., 2018). The present study evaluated the discriminant validity of the measures by employing the heterotrait-monotrait (HTMT) criteria, as suggested by Henseler et al. (2015) and Hair et al. (2018). The HTMT ratio was determined by employing the bias-corrected and accelerated (BCa) bootstrapping technique with a resample size of 3,000. A one-tailed *t*-test was conducted at a significance level of 90%. This analysis assists the researchers in obtaining estimates with a 5% error probability using the two-tailed approach (Henseler et al., 2009). According to Henseler et al. (2015), it is recommended to use a maximum threshold value of HTMT_0.85_. The findings presented in Table 1 demonstrate that all of the HTMT values are below the permissible threshold, therefore confirming the presence of discriminant validity.

**Table 1:** HTMT criterion.

	**GI**	**LM**	**LLO**	**LSP**
GI				
LM	0.601			
	CI._0.900_			
	[0.530; 0.680]			
LLO	0.631	0.643		
	CI._0.900_	CI._0.900_		
	[0.551; 0.712]	[0.576; 0.700]		
LSP	0.737	0.556	0.724	
	CI._0.900_	CI._0.900_	CI._0.900_	
	[0.672; 0.789]	[0.461; 0.643]	[0.667; 0.781]	

GI, gamification integration; LM, learners' motivation; LLO, language learning outcomes; LSP, learning style preference.

### Structural model

Upon evaluating the measurement model, this work proceeds to establish the structural model by employing a BCa bootstrapping strategy on a resample of 3,000. This approach is utilized to derive the t- and *p*-values necessary for analyzing the path coefficients (β). Additionally, the research also evaluates the coefficient of determination (*R*^2^), predictive relevance (*Q*^2^), and effect size (*f*^2^) to determine the association between the latent variables (Hair et al., 2018). Hair et al. (2018) proposed that researchers should consider calculating the effect size (*f*^2^) alongside estimating the *R*^2^ value. This additional measure allows for the evaluation of the impact of omitting a specific exogenous construct from the model on the endogenous constructs. By assessing the change in the *R*^2^ value, researchers can determine whether the omitted construct has a significant influence on the endogenous constructs (Hair et al., 2018). The findings displayed in Table 2 indicate a statistically significant and positive relationship between gamification integration and language learning outcomes (β = 0.440; *t* = 8.734; *p* = 0.000; *f*^2^ = 0.234), with a moderate effect size. The findings of the analysis provide support for Hypothesis 1.

**Table 2:** Effects on endogenous variables.

**Hypotheses**	**β**	**CI (5%, 95%)**	**SE**	***t*-value**	***p*-value**	**Decision**	***f* ^2^**	***R* ^2^**	***Q* ^2^**
*H1* GI → LLO	0.440^**^	(0.378, 0.522)	0.042	8.734	0.000	Supported	0.234	0.549	0.334
*H3* GI x LSP → LLO	0.517^**^	(0.435, 0.585)	0.050	10.483	0.000	Supported	0.145		

GI, gamification integration; LLO, language learning outcomes; LSP, learning style preference.

^**^Significance *p* < 0.05 (1.96).

Moreover, the present study employs the two-stage approach proposed by Henseler and Fassot (2010) to evaluate the moderation analysis. This approach is utilized to analyze the interaction effect of gamification integration and learning style preference on language learning outcomes. The study used BCa bootstrapping with a total of 3,000 resamples to quantify the effect magnitude. The findings presented in Table 2 indicate that the interaction term: gamification integration_learning style preference, has a statistically significant impact on language learning outcomes (β = 0.517; *t* = 10.483; *p* = 0.000; *f*^2^ = 0.145). The findings indicate that there is a moderate effect size when considering the impact of the interaction term on language learning outcomes. The findings of the analysis provide support for Hypothesis 3.

**Figure 2:**
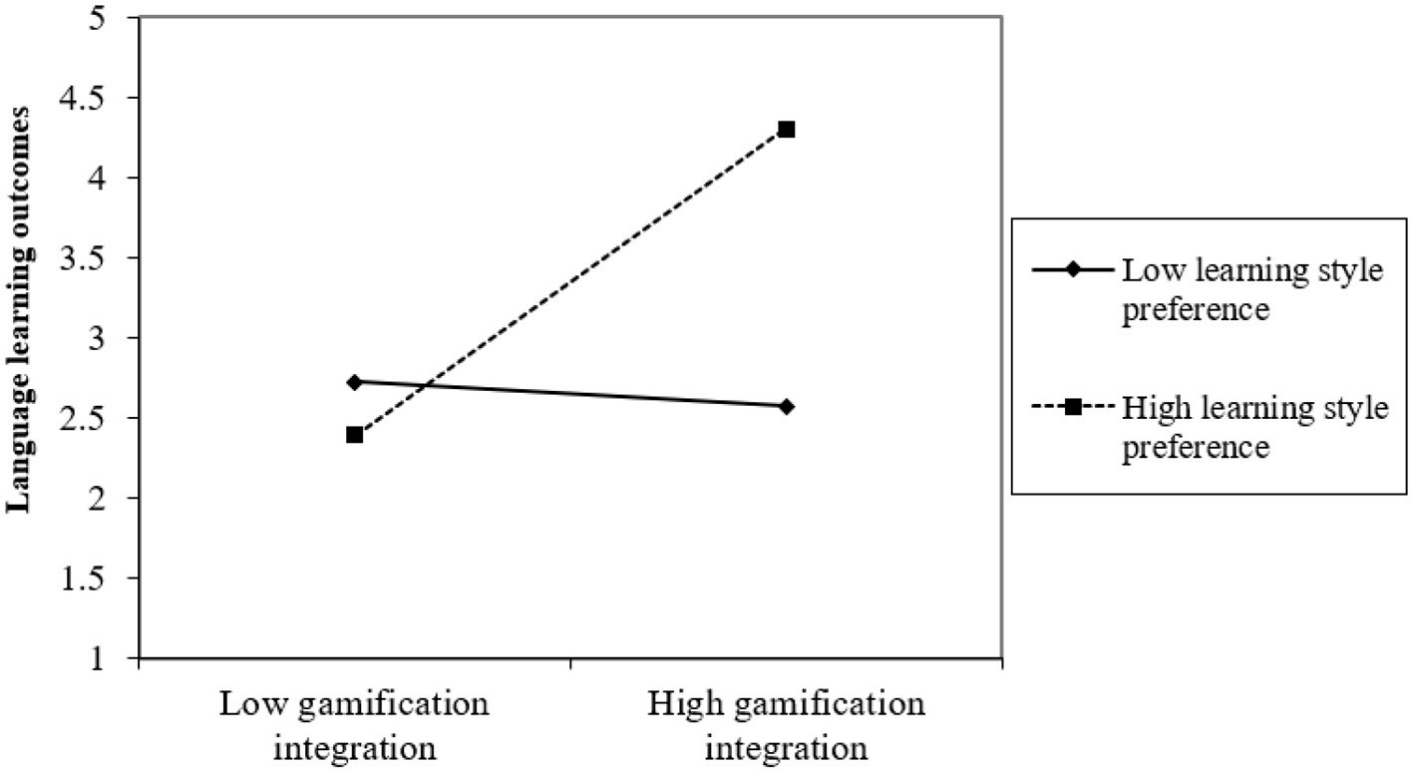
Interaction effect.

In addition, the present study evaluates the graphical depiction of the interaction effect by employing a 2-way unstandardized methodology to quantify the impact of gamification integration_learning style preference on language learning outcomes, as proposed by Dawson (2014). The findings depicted in Figure 2 indicate that when learning style preference is elevated, there exists a more robust correlation between gamification integration and language learning outcomes and vice versa.

Furthermore, the research posited that learners' motivation serves as a mediator in the relationship between gamification integration and language learning outcomes. This study evaluates the mediation analysis employing the mediation approach proposed by Zhao et al. (2010). To obtain the *t*- and *p*-values, the study conducted BCa bootstrapping with 3,000 resamples. The findings displayed in Table 3 indicate that there is a statistically significant direct impact of gamification integration on language learning outcomes, as evidenced by the confidence intervals (0.252, 0.410). Furthermore, the indirect effect of gamification integration on language learning outcomes through learners' motivation is significant with CIs (0.212, 0.376), indicating complementary mediation (Hair et al., 2018). Furthermore, the investigation also evaluated the variance accounted for (VAF). The analysis produced a VAF value of 45.83%, indicating that learners' motivation partially mediates the relationship between gamification integration and language learning outcomes. This finding provides support for Hypothesis 2. Finally, the study utilized Stone-Geisser's (*Q*^2^) method with an omission distance of 5 to assess the predictive relevance. The presence of a positive *Q*^2^-value confirms the predictive validity of the proposed model.

**Table 3:** Summary of mediating effect tests.

	**Path**	***t*-value**	**BCCI**		**Path**	***t*-value**	**95% BCCI**	**Decision**	**VAF**
Direct effect				Indirect effect					
GI → LLO	0.338^**^	12.418	(0.252, 0.410)	GI → LM → LLO	0.286^**^	8.340	(0.212, 0.376)	Supported	45.83%

GI, gamification integration; LM, learners' motivation; LLO, language learning outcomes.

^**^Significance *p* < 0.05 (1.96).

## Discussion

The current study draws on the SDT (Deci and Ryan, 1985) and ART (Kaplan et al., 1989) to investigate the influence of gamification integration on language learning outcomes through the mediating influence of learners' motivation and the moderating effect of language style preference. To test the hypothesized relationships, the study collects data from Chinese students enrolled in linguistic programs and analyzes using SmartPLS SEM. Our empirical data confirms all the proposed associations such that (1) gamification integration significantly enhances language learning outcomes, (2) leaners' motivation mediates the linkage between gamification integration and language learning outcomes, and (3) learning style preference moderates the association between gamification integration and language learning outcomes through learners' motivation, i.e., at high levels of learning style preference, the association between gamification integration and language learning outcomes is more potent and vice versa. These findings present substantial theoretical and practical implications in several ways.

The integration of gamification within the language learning context appears to resonate strongly with the core tenets of SDT, particularly in terms of fostering intrinsic motivation, autonomy, and relatedness (Ryan and Deci, 2017). Gamification, by its very nature, offers elements of choice, challenge, and feedback, aligning with the need for autonomy and competence as outlined in SDT (Ryan and Deci, 2022). This alignment is crucial in understanding how gamification elements can be more effectively designed to cater to these psychological needs, thereby enhancing intrinsic motivation among learners.

Furthermore, the role of learning style preferences as a moderator in this study suggests a nuanced understanding of individual differences in the learning process. This finding aligns with the emphasis on personal relevance and learner autonomy in SDT, indicating that gamification strategies which align with individual learning preferences are likely to be more effective (Deci and Ryan, 2012).

In expanding upon these theoretical connections, future research could benefit from further exploring how specific elements of gamification cater to the different aspects of intrinsic motivation as defined in SDT. For instance, studies could investigate how the design of gamified language learning experiences can support a sense of relatedness among learners, a key component of SDT that has been less emphasized in gamification research (Deci and Ryan, 2013).

### Theoretical implications

The study presents a multitude of theoretical implications. The study initially postulated a significant relationship between gamification integration and language learning outcomes, anticipating that the incorporation of game elements would positively influence learners' language proficiency and skills. The empirical data validate this hypothesis, highlighting that the extent of gamification integration significantly impacts language learning outcomes among Chinese students. This finding is theoretically significant as it reinforces the existing literature emphasizing the motivational and engagement-enhancing potential of gamification in language education (Noels et al., 2019; Li et al., 2022). Moreover, it aligns with recent research by Ishaq et al. (2021), which emphasized the direct impact of gamified language learning platforms on language proficiency. Thus, the study advances the current line of inquiry on gamification's effectiveness in language learning, emphasizing its role in improving language outcomes.

The second hypothesized relationship posited that learners' motivation mediates the association between gamification integration and language learning outcomes. Our findings confirm this relationship, indicating that learners' motivation plays a crucial intermediary role in translating gamification's influence into improved language proficiency. This finding holds theoretical significance by substantiating the implications of SDT (Deci and Ryan, 1985) within gamified language learning environments. It aligns with previous research that found intrinsic motivational benefits of gamification (Chan et al., 2018) and furthers the implications of SDT as a robust theoretical framework for understanding the motivational dynamics in language education. These findings are also congruent with recent studies emphasizing motivation's meaningful role in language learning (Treiblmaier and Putz, 2020), thereby reinforcing the theoretical underpinnings of this relationship.

The third hypothesis proposed a moderating effect of learning style preference on the relationship between gamification integration and language learning outcomes, suggesting that the impact of gamification varies with different learning styles. Empirical data confirm this moderating influence, such that the association between gamification and language learning outcomes is more pronounced when learners' preferences align with the gamified elements. This result extends the theoretical discourse by encompassing the ART (Kaplan et al., 1989) as a theoretical underpinning to understand the influence of individual cognitive responses to gamified environments. Theoretically, this finding enriches the existing literature by emphasizing the importance of personalized pedagogical approaches in gamified language learning. To the best of authors' knowledge, these relationships are hitherto underexplored in prior research. Thus, our study complements prior research emphasizing the value of individualized learning experiences (Kompen et al., 2019), thereby contributing to a more nuanced understanding of the interplay between gamification, cognitive responses, and language learning outcomes.

Moreover, in comparison to previous research, our study aligns with the broader consensus on gamification's positive impact on motivation and learning outcomes. However, extending the boundary conditions by investigating learning style preference as a moderating variable and learners' motivation as a mediating variable expands the theoretical landscape, addressing the need to consider individual cognitive responses in gamified language learning platform design. While prior research acknowledged the importance of individual differences in learning preferences (Li, 2022), the current study explicitly demonstrates how aligning gamification with these preferences can enhance the effectiveness of language learning. Besides, our study enriches the theoretical literature on personalized learning experiences (Xie et al., 2019) and accentuates the value of considering individual cognitive responses when designing gamified language learning platforms.

### Practical implications

The findings of this study offer several practical implications for educators, instructional designers, and language learning practitioners. First, relationship between gamification integration and language learning outcomes stresses the significance of incorporating gamified elements into language learning platforms. Educators can consider integrating game-like features such as points, leader boards, and rewards to enhance student engagement and motivation. By doing so, they can create more dynamic and captivating language learning environments that encourage active participation and, consequently, improve language proficiency and performance.

Second, the mediating role of learners' motivation in the relationship between gamification and language learning outcomes highlights the importance of fostering intrinsic motivation. We suggest that educational institutions should harness gamification not only as a tool for enhancing language skills but also as a means to cultivate students' genuine interest in language learning. In this regard, they can implement strategies such as providing autonomy in selecting learning activities and offering meaningful challenges in order to help nurture their intrinsic motivation. Additional practical approaches might include gamified language assignments, interactive quizzes, and narrative-driven language exercises that tap into learners' intrinsic curiosity and passion for the language.

Furthermore, the influence of learning style preference on the outcome of language learning indicates that tailoring language learning experiences to accord with individual preferences can result in improved results. It is imperative for language educators and policymakers to take into account the customization of gamified tasks in order to accommodate a wide range of learning methods, including visual, auditory, and kinesthetic inclinations. For example, individuals who possess a preference for visual learning may have advantages while engaging in gamified activities that incorporate visually appealing visuals. Conversely, those who exhibit a preference for aural learning may excel in environments that incorporate audio components. By taking into consideration these variations, instructors have the ability to establish a language learning experience that is both comprehensive and efficient.

Additionally, the results of the study emphasize the possibility of incorporating individualized gamified components into massive open online courses (MOOCs) and other online platforms designed for language learning. By using these institutions, the advantages of gamification can be expanded to a broader demographic, allowing individuals from various backgrounds and geographical places to partake in immersive language instruction. Additionally, instructors of MOOCs have the opportunity to investigate methods for integrating tailored gamified modules that can adjust to the preferences and proficiency levels of learners. This has the potential to improve the overall efficacy and inclusivity of language learning.

Moreover, the practical ramifications encompass the domain of language assessment and evaluation. From this standpoint, it is possible to incorporate gamified components into language competence examinations and assessments in order to replicate authentic language usage situations. Last, language educators might utilize the findings from this study to broaden the range of instructional methodologies they employ. Educators can enhance the flexibility and adaptability of the educational environment by acknowledging the influence of gamification and the significance of individual learning preferences. By employing this method, educators can provide students with a diverse range of gamified activities that correspond to various learning styles, so enabling them to select the strategies that resonate most effectively with their individual preferences.

### Limitations and future directions

Although this study has provided significant contributions to the understanding of the connection between gamification, motivation, learning style preference, and language learning outcomes, it is important to acknowledge its inherent limitations. The primary focus of the study was on Chinese students enrolled in linguistic programs, which restricts the applicability of the results to a broader and more diversified international community of language learners. In order to establish the wider applicability of these findings, future studies should aim to include a more diverse and varied population.

Furthermore, the present study utilized self-report measures to assess learning style preference, motivation, and other constructs. It is important to acknowledge that such measures are prone to response bias and social desirability effects. Therefore, subsequent investigations could potentially integrate additional objective measures or amalgamate self-report data with behavioral assessments in order to augment the validity of the findings.

Another constraint is the utilization of cross-sectional data, hence constraining the capacity to establish causal relationships. Hence, longitudinal studies should offer the potential to enhance comprehension of the intricate relationship between gamification, motivation, learning style preference, and language learning results over an extended period.

Finally, although this study examined the moderating effect of learning style choice within the gamification setting, it did not investigate the possible impact of additional individual variances, such as past language learning experience or cultural background. To enhance the comprehensiveness of the understanding regarding the correlation between personal attributes and gamified language acquisition, it is recommended that forthcoming studies take into account these elements.

## Conclusion

In conclusion, this study provides a comprehensive examination of the impact of gamification on language learning outcomes, particularly focusing on Chinese students. By integrating theories such as Self-Determination Theory and Attention Restoration Theory, and employing a robust methodology, the research offers valuable insights into the interplay between gamification, learner motivation, and individual learning styles. The findings highlight the significance of personalized gamification approaches in enhancing language proficiency, highlighting the critical role of intrinsic motivation and learning preferences. This research not only contributes to the theoretical understanding of gamified language learning but also offers practical implications for educators and curriculum designers in creating more effective and engaging learning environments.

## Data availability statement

The datasets presented in this article are not readily available because, the data and material used in this article is confidential and may be requested by the corresponding author. Requests to access the datasets should be directed to ML, minjie_lai@gcc.edu.cn.

## Ethics statement

This study adheres to the Guidelines of the ethical review process of the Quality Enhancement Cell (QEC), The University of Faisalabad, Faisalabad, Pakistan. The respondents' consent was also sought for their participation in this study. The studies were conducted in accordance with the local legislation and institutional requirements. Written informed consent for participation was not required from the participants or the participants' legal guardians/next of kin because, all the data is confidential and requested by the corresponding author. The data do not involve any collection of plants, animals, or other material from a natural setting. Further, the data is analyzed anonymously.

## Author contributions

ZS: Formal analysis, Methodology, Validation, Visualization, Writing—original draft. ML: Conceptualization, Data curation, Formal analysis, Methodology, Writing—original draft. FW: Conceptualization, Data curation, Investigation, Methodology, Writing—original draft, Writing—review & editing.
