# Supplementary material for: Investigating the influence of gamification on motivation and learning outcomes in online language learning
Source: Front Psychol. 2024 May 27;15:1295709. doi: 10.3389/fpsyg.2024.1295709 (PMC11163042; doi:10.3389/fpsyg.2024.1295709)
Supplement: Supplementary file 1 [file Data_Sheet_1.docx]

**Appendix I**

**Research instrument**

| Table 1. Validity and reliability of research instrument | | | | |
| --- | --- | --- | --- | --- |
|  | Loadings | AVE | CR | Alpha |
| *Gamification integration* |  | 0.522 | 0.812 | 0.768 |
| "Rate how critically you think when learning with these features." | 0.742 |  |  |  |
| "Assess your level of critical thinking when achieving or not achieving badges." | 0.654 |  |  |  |
| "Evaluate how critically you think about the impact of leaderboards on your language learning." | 0.831 |  |  |  |
| "Consider how critically you reflect on the motivational effect of rewards in the platform." | 0.705 |  |  |  |
| "Determine how critically you analyze the interactivity of the platform and its influence on your language learning." | 0.745 |  |  |  |
| "Rate your level of critical thinking regarding the immediate feedback you receive from gamified tasks." | 0.695 |  |  |  |
| "Assess how critically you consider the narrative elements within the gamification system in developing your language skills." | 0.682 |  |  |  |
| "Evaluate the extent to which you critically think about the challenge level of tasks and its impact on your learning." | 0.782 |  |  |  |
| "Reflect on how critically you assess the role of peer collaboration in the gamified environment for language learning." | 0.604 |  |  |  |
| "Consider how critically you evaluate the variety and effectiveness of quizzes and exercises in the platform." | 0.727 |  |  |  |
| "Determine your level of critical analysis regarding the effectiveness of time-bound challenges in the system." | 0.784 |  |  |  |
| "Rate how critically you assess the variety of tasks in the gamification platform and their role in language learning." | 0.683 |  |  |  |
| "Evaluate how critically you think about the balance between entertainment and education in the gamified learning experience." | 0.733 |  |  |  |
| *Learners’ motivation* |  | 0.543 | 0.885 | 0.824 |
| "I engage in my language studies because I find them interesting and enjoyable." | 0.634 |  |  |  |
| "I study the language to meet my personal goals and aspirations." | 0.664 |  |  |  |
| "I put effort into learning the language because I want to achieve high grades or external rewards." | 0.821 |  |  |  |
| "I learn the language because I feel it's important to fulfill the expectations of my teachers or family." | 0.764 |  |  |  |
| "I study the language because I feel I should, even though I may not always enjoy it." | 0.787 |  |  |  |
| *Language learning outcomes* |  | 0.600 | 0.901 | 0.876 |
| "I am not proficient in speaking the target language." | 0.734 |  |  |  |
| "My speaking proficiency in the target language is somewhat limited." | 0.812 |  |  |  |
| "I consider myself moderately proficient in speaking the target language." | 0.775 |  |  |  |
| "I am proficient in speaking the target language." | 0.643 |  |  |  |
| "I am very proficient in speaking the target language." | 0.824 |  |  |  |
| "I can communicate effectively and fluently in the target language in a variety of contexts and situations." | 0.842 |  |  |  |
| *Learning style preference* |  | 0.601 | 0.898 | 0.848 |
| "I prefer listening to lectures rather than reading textbooks." | 0.738 |  |  |  |
| "I prefer learning by doing exercises and drills in the class." | 0.764 |  |  |  |
| "I find that I learn better when information is presented visually, such as through charts or diagrams." | 0.736 |  |  |  |
| "I understand concepts more easily when they are explained in a written format." | 0.722 |  |  |  |
| "Group discussions and collaborative learning are more effective for me than solo study." | 0.800 |  |  |  |
| "I retain information better when I can apply it in practical, real-world situations." | 0.821 |  |  |  |
| "I prefer learning through hands-on activities and experiments." | 0.781 |  |  |  |
| "Watching videos or multimedia presentations helps me understand new concepts better." | 0.762 |  |  |  |
| "I find it easier to remember information when it’s presented through storytelling or narrative formats." | 0.803 |  |  |  |
| "I prefer step-by-step instructions and structured learning environments." | 0.748 |  |  |  |
| "I learn more effectively when I can debate and discuss topics with others." | 0.792 |  |  |  |
| "I benefit from learning in a quiet, individual study environment." | 0.833 |  |  |  |
| "I grasp new languages or concepts better through auditory methods like listening to recordings or lectures." | 0.764 |  |  |  |
| *Notes.* GI: gamification integration; LM: learners’ motivation; LLO: language learning outcomes; LSP: learning style preference | | | | |
